# ABC transporters are involved in defense against permethrin insecticide in the malaria vector *Anopheles stephensi*

**DOI:** 10.1186/1756-3305-7-349

**Published:** 2014-07-29

**Authors:** Sara Epis, Daniele Porretta, Valentina Mastrantonio, Francesco Comandatore, Davide Sassera, Paolo Rossi, Claudia Cafarchia, Domenico Otranto, Guido Favia, Claudio Genchi, Claudio Bandi, Sandra Urbanelli

**Affiliations:** Department of Veterinary Science and Public Health, University of Milan, Milan, Italy; Department of Environmental Biology, University “La Sapienza” of Rome, Rome, Italy; Department of Biology and Biotechnology, University of Pavia, Pavia, Italy; School of Bioscience and Veterinary Medicine, University of Camerino, Camerino, Italy; Department of Veterinary Medicine, University of Bari, Bari, Italy

**Keywords:** Mosquitoes, Bioassays, Insecticide resistance, Culicidae, Vector control, ABC transporters

## Abstract

**Background:**

Proteins from the ABC family (ATP-binding cassette) represent the largest known group of efflux pumps, responsible for transporting specific molecules across lipid membranes in both prokaryotic and eukaryotic organisms. In arthropods they have been shown to play a role in insecticide defense/resistance. The presence of ABC transporters and their possible association with insecticide transport have not yet been investigated in the mosquito *Anopheles stephensi,* the major vector of human malaria in the Middle East and South Asian regions. Here we investigated the presence and role of ABCs in transport of permethrin insecticide in a susceptible strain of this mosquito species.

**Methods:**

To identify ABC transporter genes we obtained a transcriptome from untreated larvae of *An. stephensi* and then compared it with the annotated transcriptome of *Anopheles gambiae.* To analyse the association between ABC transporters and permethrin we conducted bioassays with permethrin alone and in combination with an ABC inhibitor, and then we investigated expression profiles of the identified genes in larvae exposed to permethrin.

**Results:**

Bioassays showed an increased mortality of mosquitoes when permethrin was used in combination with the ABC-transporter inhibitor. Genes for ABC transporters were detected in the transcriptome, and five were selected (*Anst*ABCB2, *Anst*ABCB3, *Anst*ABCB4, *Anst*ABCmember6 and *Anst*ABCG4). An increased expression in one of them (*Anst*ABCG4) was observed in larvae exposed to the LD50 dose of permethrin. Contrary to what was found in other insect species, no up-regulation was observed in the *Anst*ABCB genes.

**Conclusions:**

Our results show for the first time the involvement of ABC transporters in larval defense against permethrin in *An. stephensi* and, more in general, confirm the role of ABC transporters in insecticide defense. The differences observed with previous studies highlight the need of further research as, despite the growing number of studies on ABC transporters in insects, the heterogeneity of the results available at present does not allow us to infer general trends in ABC transporter-insecticide interactions.

**Electronic supplementary material:**

The online version of this article (doi:10.1186/1756-3305-7-349) contains supplementary material, which is available to authorized users.

## Background

Malaria is a major threat to human health and socio-economic development, representing a great burden in the vast regions of the world in which this parasitosis is endemic [1–3]. WHO estimated over 200 million cases of malaria in the 99 endemic countries and around 660,000 deaths, in the year 2010 [2].

Vector control through insecticides is a core component of malaria control programmes. However, continuous use of insecticides has led to the development of resistance in many malaria vectors around the world, which poses a serious threat to the global malaria control efforts [3–5]. Research is therefore needed to understand the molecular basis of insecticide detoxification and develop even more effective methods to delay emergence of resistance [6].

In recent years, the role of ATP-binding cassette (ABC) transporters in the defense against toxic compounds as pesticides has attracted a great deal of attention (reviewed in [7, 8]). ABC transporters are ATP-dependent efflux pumps belonging to the ABC protein family located in the cellular membrane in both prokaryotic and eukaryotic organisms. In eukaryotic organisms, they mediate the efflux of compounds from the cytoplasm to the outside of the cell or into organelles. ABC proteins have been subdivided into eight subfamilies (from ABC-A to ABC-H), and can transport a wide array of different substrates across cellular membranes (e.g., amino-acids, sugars, lipids, and peptides). Most of the ABC transporters associated with the efflux of pesticides belong to the subfamilies ABC-B (also referred to as P-glycoproteins, P-gps), ABC-C and ABC-G. In some cases ABC-transporter action has also been associated with insecticide-resistant phenotypes in species of agricultural or medical importance [7, 8]. In spite of an increasing awareness of the potential importance of ABC transporters in vector control, to date they have been poorly studied in detail in malaria vectors [8]. Here, we investigated the role of ABC transporters in the detoxification against the insecticide permethrin in the malaria vector *Anopheles stephensi* (Culicidae: Diptera). This mosquito species, vector of both *Plasmodium falciparum* and *Plasmodium vivax*, is one of the major vectors of human malaria in the world. *An. stephensi* occupies a geographic range that spans from the Middle East to South-East Asia [9]. These regions contribute to 15% of malaria cases worldwide, with an estimated 28 million people annually affected by the disease [2]. Permethrin belongs to the pyrethroid class of insecticides, which is by far the most commonly used in malaria vector-control interventions [2].

Pyrethroids act by modifying the gating kinetics of voltage-gated sodium channels, thereby disrupting neuron function, which leads to rapid paralysis and death of the insect [10]. They can enter into the insect body by ingestion and penetration into the hemolymph through the alimentary canal, or via contact with sensory organs of the peripheral nervous system [11]. Insect midgut is rich in ABC transporters, whose action, therefore, likely prevents permethrin to reach its target sites [8]. Furthermore, insects possess protective neural barriers (e.g. a layer of glially derived epithelial cells), where ABC transporters likely play an important role in the exchange of molecules [12, 13]. In particular, inhibition of P-gp in *Schistocerca gregaria* has been shown to increase brain uptake of different drugs [13]. The involvement of ABC transporters in pyrethroid detoxification has been reported for a few insect species, such as *Helicoverpa armigera* [14–16], *Apis mellifera* [17] and *Culex pipiens* [18]. Up-regulation of ABC-transporter genes has also been reported in pyrethroid resistant strains of the bed bugs *Cimex lectularius* [19] and of the vector mosquitoes *Anopheles gambiae* [20] and *Aedes aegypti* [21]. No protein belonging to the ABC transporters has yet been described in larvae of *An. stephensi*, nor the possible association of this class of proteins with insecticide transport has been investigated in this species. In this paper we investigated the presence and role of ABCs in transport of permethrin insecticide in larvae of *An. stephensi*: *i*) by bioassays with permethrin alone and in combination with an ABC inhibitor; *ii*) by investigating gene expression profiles in larvae exposed to permethrin treatment.

## Methods

### Mosquito samples

The mosquito larvae used in this study were obtained from adult females of a *An. stephensi* colony, derived from the Liston strain. This colony has been maintained for four years in the insectary at the University of Camerino, following standard conditions: adult insects are reared at 28 ± 1°C and 85-90% relative humidity with photoperiods (12:12 L-D) with a 5% sucrose solution, and adult females are fed with mouse blood for egg laying. Eggs from this colony were put into spring water in order to obtain the larvae. Larvae were maintained in spring water and fed daily with fish food (Tetra, Melle, Germany) under the same conditions as the adults.

### Bioassays

Inhibition of ABC-transporters should lead to a higher intracellular concentration of insecticide, thus increasing larval susceptibility and insecticide efficacy [8]. In order to evaluate a potential synergy, we performed bioassays with permethrin insecticide alone and with permethrin in combination with a sub-lethal dose of the ABC-transporter inhibitor verapamil (see below for experimental determination of sub-lethal dose of verapamil). This is a calcium channel blocker, which works by competing with cytotoxic compounds for efflux by the membrane pumps [22]. All bioassays were conducted on *An. stephensi* larvae at the third instar, according to standard protocols [23].

Groups of 25 larvae were put in 250 ml plastic glasses with 100 ml of spring water and different concentrations of insecticide or insecticide + inhibitor. All tests were performed in quadruple. Additional groups of larvae, treated only with water and acetone (that was used to dilute permethrin), were used as controls. Mortality was assessed at 24 h post-treatment and the larvae were considered dead if immobile, even after a mechanical stimulus.

In the bioassays with permethrin alone (Sigma-Aldrich S.r.l., Milan, Italy), six insecticide concentrations were used (0.015, 0.047, 0.092, 0.23, 0.57, 1.44 mg/l) to have mortality in the range 1–99%. The drug was dissolved in acetone and then diluted in water to obtain the test solutions. The bioassays with permethrin in combination with verapamil were performed using permethrin at the six concentrations indicated above, plus two additional concentrations (0.0024 and 0.0048 mg/l). The sub-lethal dose of verapamil (i.e. the dose at which no dead larvae were observed) was determined using ten different concentrations (20, 40, 80, 100, 160, 240, 320, 400, 480, 560 μM) following the protocol above. The larval mortality data were subjected to Probit regression analysis [24] as implemented in the XLSTAT-Dose software (available at: http://www.xlstat.com) to estimate the LD50 values and their 95% confidence intervals (CIs). To estimate the effect on larval mortality of the ABC inhibitor at sub-lethal dose, the synergistic factor (SF) was calculated.

### Identification of ABC transporter genes

A total of 200 untreated larvae of *An. stephensi* at the third instar were pooled in 15 ml of RNAlater stabilization solution (Qiagen, Hilden, Germany) and provided to an external company (GATC Biotech AG, Costance, Germany) for one run of 2x250 paired-ends reads sequencing on the Illumina MiSeq platform. The resulting reads were assembled using Trinity with default settings [25]. The assembled contigs were compared with Blastx (evalue 0.00001) to the annotated transcriptome of *An. gambiae* available in the VectorBase database, and the sequences of ABC transporters were extracted automatically and manually controlled. Based on published results about the involvement of ABCs on multidrug resistance in several arthropods (mainly mosquitoes) [8], we selected five genes from the transcriptome of *An. stephensi.* Oligonucleotide primers were then designed from the sequence of each gene (Table 1). The sequences of ABC transporters identified in *An. stephensi* were translated to aminoacids and compared against the UniProt database [26] using Blastp. Homologous proteins were aligned using ClustalX [27] and distances among them were estimated by Dayhoff PAM matrix as implemented in the PROTDIST software of the PHYLIP package [28].

**Table 1:** **Primer sequences of ABC transporter genes identified in** ***Anopheles stephensi***

Gene	Forward primer	Reverse primer	PCR product size (base pairs)	Source
*Anst*ABCB2	TATCAAGTTCACGGATGTAGAGT	TATCCACCTTGCCACTGTC	185	This work
*Anst*ABCB3	CAACCGTTCCGTAATACTACC	ACTGGTAGCCCAATGTGAAG	133	This work
*Anst*ABCB4	GGACAAAACATTCGGGAGG	CGTAGTGAATGTTGTGGCG	109	This work
*Anst*ABCBmemb6	CTGGAGACGCTGAGAGATA	TACTCCTCGGTGAACTGG	125	This work
*Anst*ABCG4	ATGAGCCCATTCGTCCTG	AGCGTGGAGAAGAAGCAG	158	This work
Rps7	AGCAGCAGCAGCACTTGATTTG	TAAACGGCTTTCTGCGTCACCC	90	Capone et al. 2013 [31]

### Gene expression profile after insecticide treatment

The activity of ABC-transporters is generally modulated at gene transcriptional level: the presence of toxic compounds leads to higher transcription. In order to assess this topic, larvae of *An. stephensi* at the third instar were exposed to permethrin and the expression of ABC-transporter genes was monitored in the surviving larvae by quantitative RT-PCR twice after insecticide treatment: 24 h (e.g. the time at which the LD50 has been estimated) and 48 h following the study of Figueira-Mansur [29] that found increased expression of ABC transporters in the mosquito *Ae. aegypti*. The larvae were treated with the LD50 (0.137 mg/l) of insecticide estimated by bioassays as described above and two pools, of ten larvae each, were collected after 24 and 48 h of insecticide-treatment. All pools of larvae were stored in RNAlater for molecular analysis and, controls (water + acetone) were collected following the same time frame.

RNA was extracted from each pool of larvae using the RNeasy Mini Kit (Qiagen, Hilden, Germany) including an on-column DNase I treatment (Qiagen, Hilden, Germany), according to the manufacturer’s instructions. Total RNA was eluted into nuclease-free water and the concentration of RNA was determined at 260 nm [30] using a NanoDrop ND-1000 (Thermo Scientific, Delaware, USA). cDNAs were synthesized from 250 ng of total RNA using a QuantiTect Reverse Transcription Kit (Qiagen, Hilden, Germany) with random hexamers. The cDNA was used as template in RT-PCR reactions using the primers designed from the sequences of identified ABC genes (Table 1). The amplification fragments, obtained using standard PCR conditions and the thermal profile indicated below, were sequenced in order to confirm the specificity of the amplification.

Quantitative RT-PCRs on the target ABCs were performed using a BioRad iQ5 Real-Time PCR Detection System (Bio-Rad, California, USA), under the following conditions: 50 ng cDNA; 300 nM of forward and reverse primers; 98°C for 30 sec, 40 cycles of 98°C for 15 sec, 59°C for 30 sec; fluorescence acquisition at the end of each cycle; melting curve analysis after the last cycle. The cycle threshold (Ct) values were determined for each gene, in order to calculate gene expression levels of target genes relative to *rps7,* the internal reference gene for *An. stephensi* [31]. The expression of the ABC transporters genes in the control group was considered as the basal level (equal to 1). The estimates of the expression level of each gene in the treated larvae are reported as the means ± standard deviation (SD) in Additional file 1: Table S1.

### Ethical statement

Maintenance of the mosquito colony of *An. stephensi* was carried out according the Italian Directive 116 of 10/27/92 on the “use and protection of laboratory animals” and in adherence with the European regulation (86/609) of 11/24/86 (licence no. 125/94A, issued by the Italian Ministry of Health).

## Results

### Bioassays

No mortality was observed when larvae were exposed at concentrations of verapamil up to 100 μM; this concentration was thus used as the sub-lethal dose in the bioassays with insecticide + ABC-transporter inhibitor. The results of toxicity assays using permethrin and permethrin in combination with verapamil are reported in Table 2. The mortality data observed in bioassays well fitted the Probit dose–response model (Chi-Square probability <0.0001). The LD50 dose in permethrin assay was 0.137 mg/l while in the assay in combination with verapamil LD50 was 0.025 mg/l (Table 2). No overlapping values were observed between LD50 95% CI of insecticide alone and insecticide plus verapamil; the addition of verapamil increased the toxicity of permethrin about 5-fold (SF = 5.48).

**Table 2:** **Toxicity of verapamil and permethrin against** ***Anopheles stephensi*** **larvae**

Insecticide	N	Slope ± SE	LD50 (95% CI)	SF
Verapamil	600	3.846 (0.374)*	528 μM (486-587)	
Permethrin	600	1.819 (0.125)*	0.137 mg/l (0.117-0.160)	
Permethrin + verapamil (100 μM)	600	2.123 (0.174)*	0.025 mg/l (0.021-0.029)	5.48

LD50 and slopes of regression lines estimated from mortality data by Probit analysis are shown. N, number of larvae used in bioassays; SE, standard error; 95% CI, 95% confidence interval. SF, synergistic factor.

*Chi-Square probability < 0.0001.

### Isolation of ABC transporter genes and expression profile after insecticide treatment

The Illumina MiSeq platform was used to sequence the cDNA library obtained from a pool of 200 larvae of *An. stephensi*, and 16,686,276 paired-ends reads were obtained. MiSeq raw data were assembled with Trinity, obtaining 40,498 contigs. The contigs containing ABC transporter genes were extracted on the basis of the annotated transcriptome of *An. gambiae* available in database. Five sequences, respectively of 3612, 2154, 2481, 2553 and 2182 base pairs, were found to share 85-94% identity with putative ABC multidrug transporters of *An. gambiae*: ABCB2 (AGAP005639) (85% identity), ABCB3 (AGAP006273) (94% identity), ABCB4 (AGAP006364) (88% identity), ABCmember6 (AGAP002278) (94% identity) and ABCG4 (AGAP001333) (85% identity). We denoted them as *Anst*ABCB2, *Anst*ABCB3, *Anst*ABCB4, *Anst*ABCmember6, *Anst*ABCG4 and we deposited them in EMBL Nucleotide Sequence Database [EMBL: LK392613 to LK392617]. The alignment of the deduced amino acidic sequences of ABC transporters identified in *An. stephensi* with sequences of homologous ABC transporters of *An. gambiae* is shown in Additional file 2: Figure S1*.* Dayhoff PAM distance estimates between the ABC transporters identified in *An. stephensi* and the homologous ABC transporters of mosquitoes *An. gambiae, Anopheles darlingi, Ae. aegypti* and *Culex quinquefasciatus* that showed the highest percentage of identity following Blast search are shown in Additional file 3: Table S2.

**Figure 1:**
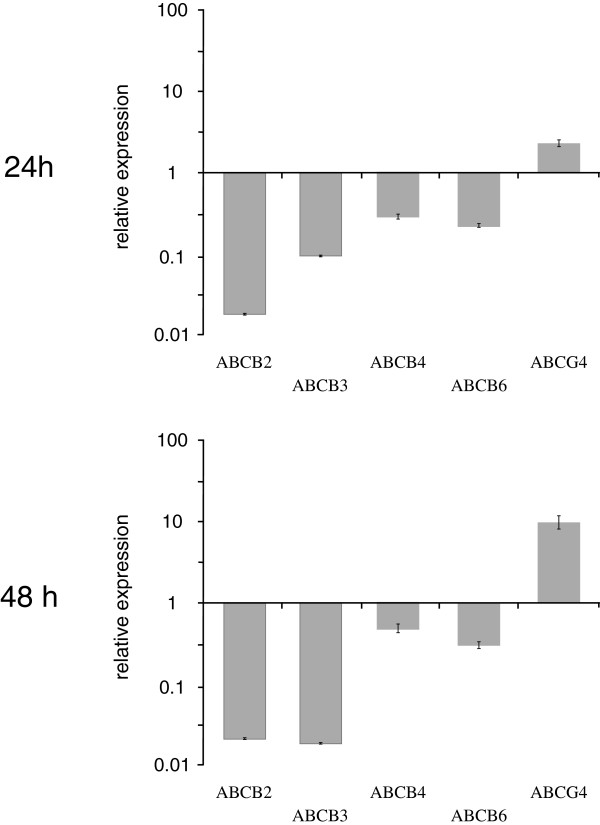
**Relative expression of** ***Anopheles stephensi*** **ABC genes measured by quantitative PCR after 24 and 48 h of permethrin exposure.** The expression level in non-treated larvae was considered to be the basal level (equal 1). The internal reference gene *rps7* for *An. stephensi* was used to normalize expression levels. The values are expressed as means ± standard deviations.

Conventional PCR amplicons obtained from each gene primer set were sequenced, confirming in all cases the sequences generated with the MiSEQ experiment*.* The RT-PCRs were performed to investigate whether permethrin treatment at the LD50 dose (0.137 mg/l, Table 2) increased or decreased the ABC gene expression in the *An. stephensi* larvae after 24 and 48 h of insecticide-treatment. As reported in Figure 1 and Additional file 1: Table S1, after 24 h of permethrin treatment, the relative expression of all selected genes was down-regulated, with the exception of the *Anst*ABCG4 gene, that showed about three-fold increase of expression compared to the control. Similarly, after 48 h of permethrin treatment, the relative expression of all ABCB genes was down-regulated, while the *Anst*ABCG4 gene showed a ten-fold increase of expression compared to the control.

## Discussion

Bioassays and molecular data suggest the involvement of ABC transporters in the defense of *An. stephensi* larvae against the permethrin insecticide. Indeed, inhibition of ABC-transporters led to a higher susceptibility of larvae to insecticide, indicating that ABC transporters are associated with insecticide detoxification. In addition, in mosquito larvae exposed to the LD50 dose of permethrin, we observed an increased expression of *Anst*ABCG4, one of the five tested genes coding for ABC transporters.

Arthropod ABCG genes are orthologous of the human gene *ABCG2*, which has been associated with resistance to cancer drugs [32, 33], while data in insects show that *ABCG* transporter genes were significantly over-transcribed in response to exposure to insecticides. Microarray gene expression studies revealed that ABCG transporter genes were up-regulated in DDT resistant strains of *Drosophila melanogaster* [34] and in a *Plutella xylostella* (Lepidoptera) strain resistant to chlorpyrifos [35]. The ABCG4 transporter gene was over-transcribed in *Bemisia tabaci* whiteflies resistant to the neonicotinoid thiamethoxam [36] as well as in *Anopheles arabiensis* resistant- and sensible-strains to DDT [33]. The ABCG3 gene was found differentially expressed in *Ae. aegypti* pyrethroid resistant populations versus susceptible strains [21].

The other four genes coding for ABC transporters that we detected and tested in *An. stephensi* (*Anst*ABCB2, *Anst*ABCB3, *Anst*ABCB4 and *Anst*ABCmember6) belong to the ABCB subfamily. Several members of this subfamily have been associated with transport and/or resistance to different insecticide classes in several insect species [7, 8]. In mosquitoes, the ABCB2 gene was showed by quantitative PCR to be eightfold up-regulated in larvae of a susceptible strain of *Ae. aegypti* analysed at 48 h post temephos-treatment [29]. The ABCB4 gene was showed to be over-transcribed by transcriptome and quantitative PCR analyses, in DDT and pyrethroid (permethrin and deltamethrin) resistant *Ae. aegypti* populations compared to a laboratory susceptible population [21]. Microarray analysis showed that the ABCB4 was up-regulated in different populations of DDT-resistant *An. gambiae* mosquitoes [37]. Our results showed no over-transcription of the ABCB genes in *An. stephensi* susceptible larvae exposed to permethrin, while insecticide treatment induced an increased expression of the *Anst*ABCG4 (Figure 1, Additional file 1: Table S1).

On the whole, the results herein presented support the view of the involvement of ABC transporters in insecticide transport, although differences with previous studies have been observed. Are these differences due to the insecticides used or to the status of the analysed samples (i.e. susceptible vs. resistant)? Despite the growing number of studies on ABC transporters in insects, the heterogeneity of the data available at present does not allow to infer general trends that may underlie particular interactions between ABC transporters and insecticides. Further studies are needed to highlight these and other issues. For example, our results showed that the expression of ABCB genes in *An. stephensi* did not only increase in larvae treated with permethrin compared to those non-treated, but indeed it decreased (Figure 1, Additional file 1: Table S1). In the latter case, the study of gene expression at more time points could contribute to the understanding of whether there are temporal delays, or whether compound-specific or species-specific differences exist in their activation [38–40]. Furthermore, most studies have been conducted on larval stages [7, 8]. The synergist and transcript profiles may differ between larval and adult stages, an interesting topic for future studies.

Diffusion of vectors of human diseases driven by human activities and global climate change as well as insurgence of insecticide resistance can seriously impact our ability to control vector-borne diseases [41–44]. Furthermore, environmental pollution and resistance phenomena clearly show the limits of the chemical approach for pest control and the need to delineate new strategies that optimize the use of available molecules, with the aim of reducing their impact on the environment [31, 45–48].

In the last decades advances in molecular techniques have greatly improved our tools to investigate the dynamics of vector populations and of pesticide resistance insurgence [43, 49–53]. More recently, next-generation sequencing technologies have offered unprecedented opportunities to investigate the molecular basis of the interaction between cellular defenses and insecticides [54]. In this context, the increasing interest about ABC transporters in transport and/or resistance against insecticides led to an increase of the information on these genes in various insect species and their association with insecticide detoxification [7, 8].

## Conclusions

In this study we have demonstrated for the first time in the larvae of *An. stephensi* that verapamil increases the sensitivity to permethrin in laboratory assays; in addition, we isolated five genes encoding for ABC transporters, and investigated their expression profile after exposure to permethrin. To analyse the potential role of ABC transporters in permethrin transport in *An. stephensi*, we performed bioassays using a sub-lethal dose of the ABC transporter inhibitor verapamil in association with permethrin. The results obtained using this approach highlight that the combination of insecticides with an ABC-transporter inhibitor can increase the efficacy of the insecticide molecule [18, 29, 55]. In prospect, combined treatments of insecticide plus ABC-transporter inhibitors could be proposed, with the objective of reducing the current dosages of insecticides or to prevent the development of resistance, and reduce environmental pollution [29, 56]. The implementation of such a strategy would require the availability of gene- and species-specific inhibitors in order to avoid the serious consequences that would derive from a generic inhibition of ABC-transporters in non-target organisms. The study of ABC-transporters at the gene level is therefore crucial for the understanding of both their potential role as defense mechanisms and for their inhibition for vector control purposes.

## Electronic supplementary material

Additional file 1: Table S1: Relative expression of *Anopheles stephensi* ABC genes measured by quantitative PCR after permethrin exposure. The expression level in non-treated larvae was considered to be the basal level (equal 1). The internal reference gene *rps7* for *An. stephensi* was used to normalize expression levels. The values are expressed as means ± standard deviations. (DOC 14 KB)

Additional file 2: Figure S1: Alignment by ClustalW of deduced amino acidic sequences of ABC transporters of *Anopheles stephensi* and *Anopheles gambiae*. Asterisks: conserved amino acid residues; colons: conserved substitutions; dots: semiconserved substitutions. (PDF 138 KB)

Additional file 3: Table S2: Dayhoff PAM matrix. Estimates of distance among the ABC transporters identified in *Anopheles stephensi* and the homologous ABC transporters of other mosquitoes species are shown: *An. gambiae* (AGAP005639; AGAP006273; AGAP006364; AGAP002278; AP001333), *An. darlingi* (ETN61204; ETN66919; ETN62617; ETN64062; ETN58714), *Aedes aegypti* (AAEL010379; AAEL002468; AAEL006717; AAEL008134; AAEL003703), and *Culex quinquefasciatus* (EDS44274; EDS35382; EDS29700; EDS27088; EDS37204). (DOC 15 KB)
