# Supplementary material for: ABC transporters are involved in defense against permethrin insecticide in the malaria vector Anopheles stephensi
Source: Parasit Vectors. 2014 Jul 29;7:349. doi: 10.1186/1756-3305-7-349 (PMC4124152; doi:10.1186/1756-3305-7-349)
Supplement: Supplementary file 1 — Additional file 1: Table S1: Relative expression of Anopheles stephensi ABC genes measured by quantitative PCR after permethrin exposure. The expression level in non-treated larvae was considered to be the basal level (equal 1). The internal reference gene rps7 for An. stephensi was used to normalize expression levels. The values are expressed as means ± standard deviations. (DOC 14 KB) [file 13071_2014_1536_MOESM1_ESM.doc]

**Table S1.** **Relative expression of *Anopheles stephensi* ABC genes measured by quantitative PCR after permethrin exposure.** The expression level in non-treated larvae was considered to be the basal level (equal 1). The internal reference gene rps7 for An. stephensi was used to normalize expression levels. The values are expressed as means ± standard deviations.

| **ABC transporter genes** | **24 h** | **48h** |
| --- | --- | --- |
| *Anst*ABCB2 | 0.021±0.012 | 0.029±0.023 |
| *Anst*ABCB3 | 0.103±0.031 | 0.025±0.019 |
| *Anst*ABCB4 | 0.495±0.040 | 0.707±0.117 |
| *Anst*ABCBmemb6 | 0.227±0.038 | 0.504±0.086 |
| *Anst*ABCG4 | 3.574±0.164 | 9.769±0.292 |
