# Supplementary material for: ABC transporters are involved in defense against permethrin insecticide in the malaria vector Anopheles stephensi
Source: Parasit Vectors. 2014 Jul 29;7:349. doi: 10.1186/1756-3305-7-349 (PMC4124152; doi:10.1186/1756-3305-7-349)
Supplement: Supplementary file 2 — Additional file 2: Figure S1: Alignment by ClustalW of deduced amino acidic sequences of ABC transporters of Anopheles stephensi and Anopheles gambiae. Asterisks: conserved amino acid residues; colons: conserved substitutions; dots: semiconserved substitutions. (PDF 138 KB) [file 13071_2014_1536_MOESM2_ESM.pdf]

***Anst*ABCB2 gene**

```
AnstABCB2   ------------------------NDFSRSTHVCT------------------------------------------------------CFANFPNLISD  100
AGAP005639  MTAKEYPQGNGTFQTMNLKNGNGADNMSVASHKSTKDILDVKAPTKEPVSQSVSYFKLFRFATWGEVCATLMGVLLASMASLGLPYGVILYGEFTTLLVD
                                    :::* ::* .*                                                       :.:*..*: *

AnstABCB2   YRR--------------------VNATEQENAAAIMEDAKAFGLGVVAVTILQFIFATLSVDIINRSAQKQISRIRQLFLKAVLRQDMTWYDLNSDDSFA  200
AGAP005639  RTIGIGKSTDTAILSIFGGGRVLVNATEQENAAAIMEDAKAFGLGVVAVTILQFIFATLSVDVINRSAQKQISRIRQLFLKAVLRQDMTWYDLNSDDSFA
                                   ***************************************:*************************************

AnstABCB2   VRITDDLDKLKEGIGEKLSIFTYLVMSFVISVIFSFFYGWKLTLVILSCAPIIILATAFVAKMQSTLTEKELKSYSSAGAVAEEVLGSIRTVVAFGGEKK  300
AGAP005639  VRITDDLDKLKEGIGEKLSIFTYLVMSFVISVIFSFFYGWKLTLVILSCAPIIILATAFVAKMQSTLTEKELKSYSSAGAVAEEVLGSIRTVVAFGGEKK
            ****************************************************************************************************

AnstABCB2   ELDRYRERLAGAELNGRRKGLFSGIGGGIMWFIIYCCYALAFWYGISLILEDRDKDLKDYTPAVLIIVLFGVLAGAQNLGLSSPHLEAFSTAKGSAATIF  400
AGAP005639  EADRYRERLAGAELNGRRKGLFSGIGGGIMWFIIYCCYALAFWYGISLILEDRDKDLKDYTPAVLIIVLFGVLAGAQNLGLSSPHLEAFSTAKGSAATIF
            * **************************************************************************************************

AnstABCB2   SVIDRKPEIDSLGDAGLRPKSMQGNIKFTNVCFRYPARNDVQVLQGLNLEIKTGQTVALVGPSGCGKSTCLQLIQRLYDPLSGTVTIDGTKVSELNIGWL  500
AGAP005639  SVIDRIPVIDSLGDAGLRPGSMQGNIKFSNVFFRYPARNDVQVLQGLNLEIKTGQTVALVGPSGCGKSTCLQLIQRLYDPLSGSVTIDGTKVSELNIGWL
            ***** * *********** ********:** ***************************************************:****************

AnstABCB2   RSFIGLVGQEPVLFATTIAENIRYGNPDATQSEIECAAKIANCHSFIMKLPNAYGTMIGERGAQLSGGQKQRIAIARALVRNPKILLLDEATSALDPNSE  600
AGAP005639  RSFIGLVGQEPVLFATTIAENIRYGNPDASQSEIERAAKIANCHSFITKLPNGYATMIGERGAQLSGGQKQRIAIARALVRNPKILLLDEATSALDPNSE
            *****************************:***** *********** ****.*.*********************************************

AnstABCB2   KRVQDALERASKGRTTLVVSHRLSTITNADKIVYIDKGVVMEQGTHEELMAARGLYYELVVASGSQKSADDDESVPLAPSAFSMRQESVEDGADGSDDES  700
AGAP005639  KRVQDALERASKGRTTLVVSHRLSTITNADKIVYIDKGLVMEQGTHEELMAARGLYYDLVVASGSQKTVDDDESVPMAPSALSMRQESVDDGAEASDDES
            **************************************:******************:*********:.*******:****:*******:***:.*****

AnstABCB2   DSGKSDEKNEEEQEEVYHVSVLRLLKLNSPEWHYILFGCAAAIVVGASFPAFAVLFGEMYGILSVADPAFVKQESNFYSLLFLALGLITGLGTFFQTYLF  800
AGAP005639  DSGKSDEKNEEEQEEVYHVSLMRLLKLNAPEWHYILFGCAAAIVVGASFPAFAVLFGEMYGILSVADPEYVKEESNFYSFLFLVLGLITGVGTFFQTYLF
            ********************::******:*************************************** :**:******:***.******:*********

AnstABCB2   NIAGVRLTSRLRQKSFKAIVTQDMAWFDESRNAVGALCARLSGDCASVQGATGTRIGSLLQAASTICIGVGISFFYSWNLTLVSIVAIPVTLASITLESR  900
AGAP005639  NIAGVRLTSRLRQKSFKAIVSQDMAWFDESRNAVGALCARLSGDCASVQGATGTRIGSLLQAASTICIGVGISFFYSWNLTLVSIIAIPVTLASITLESR
            ********************:*****************************************************************:**************

AnstABCB2   YTQMSSLKEKQSLEGATKLAVEAISNIRTVASLGQEQHVLHRYSQETVKIDDACRRKTRLRGTVFALGQVMPFAGYGLALFYGGKLVSEKELEYKDVIKV  1000
AGAP005639  YSQTSSLKEKQSQEGATKLAVEAISNIRTVASLGQEKHVLQRYGEETVKIDDACRRKTRLRGTVFALGQVMPFAGYGLALFYGGKLVSEKELEYKDVIKV
            *:* ******** ***********************:***:**.:*******************************************************
```

(continue)

(continue)

```
AnstABCB2    SEALIFGAWMLGQALAYAPNVNSAILSAGRLMKLLDRTPRMHNPSSSYHSLSQRTEGDIKFTDVEFRYPTRPSVPVLQGLNLDIGKGQTVALVGPSGCGK  1100
AGAP005639   SEALIFGAWMLG-ALAYAPNVNSAILSAGRLMKLLDRTPRMHNPSTSYHSLSQRTEGDIKFTDVEFRYPTRPTVPVLQGLNLDIGKGQTVALVGPSGCGK
             ************ ********************************:**************************:***************************

AnstABCB2    STCIQLLLRYYDPDSGKVDIDGTTTTEFSLNRIRAQMGLVSQEPILFDRTIAENIAYGDNTRDIAMPEIIEAAKMANIHEFIVNLPKGYDTSLGSKGAQL  1200
AGAP005639   STCIQLLLRYYDPDSGKVDIDGTTTTEFSLNRIRAQMGLVSQEPILFDRTIAENIAYGDNTREIAMPEIMEAAKMANIHEFIVNLPKGYDTSLGSKGAQL
             **************************************************************:******:******************************

AnstABCB2    SGGQKQRIAIARALVRNPRVLLLDEATSALDNQSEKIVQNALDHARTGRTCIIIAHRLTTIQNANLICVIQNGVVVEAGTHDELMAKNRIYAKLYQMQQVA 1301
AGAP005639   SGGQKQRIAIARALVRNPRVLLLDEATSALDNQSEKIVQNALDHARTGRTCIIIAHRLTTIQNANLICVIQNGVVVEAGTHDELMAKSRIYAKLYQMQQVA
             ***************************************************************************************.*************
```

AnstABCB3 gene

```
AnstABCB3   MLKLLLSPHRQCCWQGLLRSSPYSTDSKHVLVRKTLQSFGSGRTGFLQPFRNTTLR-AVRPSHTVTKTGSQGGRLIRVLLGGTAVTFTLGYQYRKLFVHC  100
AGAP006273  MLKLLLSPHRQCCWQGLTRSSPYSTEGKHVLLRKTVQSFGLGRNGFLQPIRNTIQRTVVRPSHTVTKTGSQGSRLIRVLLGGTVVSITLGYQYRKSFVHC
            ***************** *******:.****:***:**** **.*****:***  * .**************.**********.*::******** ****

AnstABCB3   EASVNRLAGQRNLLDDGTVRFDWWKFWRYLRHHLGKLIGAVLAALAVAYFNIQIPNLLGVIVNKLARYAGSSLKDINTASFFRDMRGPALQLFGMYLAQS  200
AGAP006273  EASVNRLAGQRQLVDDGAVRFDWWKFWHYLRRHLGKLIGAVMAALAVAYFNIQIPNLLGVIVNKLARYAGSSIKDINTASFFRDMRGPALQLFGMYLAQS
            ***********:*:***:*********:***:*********:******************************:***************************

AnstABCB3   GFTFLYILLLSQIGEQMAASIRQDLFKQIIIQDLEFFDHNRTGELVNRLTADVQDFKSSFKQCISQGLRSFAQLVGGGISLFWISPQLASIALVSVPLAV  300
AGAP006273  GFTFLYILLLSQIGEQMAASIRQDLFKQIIIQDLEFFDHNRTGELVNRLTADVQDFKSSFKQCISQGLRSFAQLVGGGISLFWISPQLASIALVSVPVAV
            *************************************************************************************************:**

AnstABCB3   AMFSLLGRSLRHLSKKAQAQSERATSVSEEALSNIRTVRASASEYSEVELFRAETEKSAVISQQLGAGIAVFQALSNLCLNGMVLTTLLLGGHFMSANSI  400
AGAP006273  AMFSLLGRSLRHLSKKAQAQSERATSVSEEALSNIRTVRASASEYSEIELFRVETDKSAVLSQQLGAGIAVFQALSNLCLNGMVLTTLLLGGHFMSANSI
            ***********************************************:****.**:****:***************************************

AnstABCB3   SAGDLMAFLVAAQGVQRSLAQGSILLGSVIRGMTAGARVFEFLSLEPRVDLKHGLIIPHGNLKGEIRFEGVQFVYPTRPNQRVLKDFNLVLRPGQTVALV  500
AGAP006273  SAGDLMAFLVAAQGVQRSLAQGSILLGSVIRGMTAGARVFEFLSLEPRVDLKHGLIIPDSNLRGEIRFEGVQFIYPTRPNQRVLKDFNLVLRPGQTVALV
            **********************************************************..**:**********:**************************

AnstABCB3   GASGSGKSTVAALLERFYEPTAGRITIDGYELGHLSPSWLRGELIGFIEQQPVLFGTTIYENIRYGRPNATREEVMEAAKMSQSHEFVSRLPDGYDTMVG  600
AGAP006273  GASGSGKSTVAALLERFYEPTAGRITIDGYELGHLAPSWLRGELIGFIEQQPVLFGTTIYENIRYGRPNATREEVMEAAKMSQSHEFVSRLPDGYETMVG
            ***********************************:***********************************************************:****

AnstABCB3   ERGIQLSGGQRQRIAIARALLKQPLILILDEATSALDAASESIVQSALDAAVLDRTTLVIAHRLSTIRNADVIVVLDQGQIVEVGTHDSLIKAQGYYYEL  700
AGAP006273  ERGIQLSGGQRQRIAIARALLKQPTILILDEATSALDAASEAVVQRALDAAVLDRTTLVIAHRLSTIRNADVIVVLDQGQIVEVGTHDSLLKAQGYYYEL
            ************************ ****************::** ******************************************************

AnstABCB3   VKQQERQQRQEQDSRALG  718
AGAP006273  VKQQERQQRQEHDARSYG
            ***********:*:*: *
```

AnstABCB4

```
AnstABCB4       MAALLLQVVSKQAPQLNRNLLEGVRLSYKNHHPLCENYCRLHSRQLHVSGVVVANWYNDDARSRKGGRREPWAQRRNYSTE----TKNQQATENALLNIS  100
AGAP006364      MAA-LLHVVSKQAPQLNRNLLEGVRLSFKNHHPLSETHCRLQSRQLHVSAVAVANWYHD---NRKRKHSEPWGQRRSYATQGALPEKNQQATENALLNIF
                *** **:********************:******.*.:***:*******.*.*****:*   .**  : ***.***.*:*:     *************

AnstABCB4       QKKKKAPLLLAGVSGCGSQPLQSPNPRPQQQHQEQERCNRRDDGRNTPNRLHPSSTTSSSFAGGPLATKRFIITSSGLSFLPCQLTTNSLQAAPLTKQTP  200
AGAP006364      QKKRKAPWEIRTTDSSHSATRQ----RPQAQ---QERYYRRSGDSSMTNRLHPSSFSSSMLRGPPLAKPTSAITSNGSSIL--LPATQPSLQAPFTKQPP
                ***:***  :  .... * . *    *** *   ***  **... . .******* :** : * ***.    ***.* *:*    :*:.   **:***.*

AnstABCB4       PIRHCFHLGHHPASGESIGTYDGPEITATDMIKAMATYIWPKDDAMVRKRVLISLSLLGGAKVLNVCVPFLFKMGVDNLNLLSMDTVPQAASALTISVLL  300
AGAP006364      PIRHCFHLGHHPASGESIGTYDGPEITATDMIKAMATYIWPKDDAMVRKRVLISLGLLGGAKVLNVCVPFLFKMGVDNLNVLSMDTVPQAASALTISVLL
                *******************************************************.************************:*******************

AnstABCB4       GYGIARAGAAGFNELRNAVFARVAQHSIRKIATNVFLHLHNLDLQFHLSKQTGALSKTIDRGSRGINFVLTAMVFNVVPTMFELALVSSILGVKCGMAYA  400
AGAP006364      GYGIARAGAAGFNELRNAVFARVAQHSIRKIATNVFLHLHNLDLQFHLSKQTGALSKTIDRGSRGINFVLTAMVFNVVPTMFELALVSSILGMKCGMAYA
                ********************************************************************************************:*******

AnstABCB4       ALSMGCVGVYSAYTLAVTQWRTKFRIYMNQAENEAGNKAVDSLINYETVKYFNNEQYEANRYDQVLKKYEDASLKTSTSLALLNFGQNAIFSVALSTIMV  500
AGAP006364      ALSMGCVGVYSAYTLAVTQWRTKFRIYMNQAENEAGNKAVDSLINYETVKYFNNEQYEANRYDQVLKKYEDASLKTSTSLALLNFGQNAIFSVALSTIMV
                ****************************************************************************************************

AnstABCB4       MAANEIAQGRMTVGDLVMVNGLLFQLSIPLGFLGSVYREVRQALLDMRTMFTLMGVQSAIQSRVNAPPLDVSRETASIEFRNVGFRYAQSNDIFKDLSFT  600
AGAP006364      MAANEIAQGRMTVGDLVMVNGLLFQLSIPLGFLGSVYREVRQALLDMRTMFTLMGVQSAIQSRANAPPLDVRRETASIEFRNVGFRYAQSNEIFKDLSFT
                ***************************************************************.******* *******************:********

AnstABCB4       IPAGKKIAIVGGSGSGKSSMVRLLYRFFEPTTGEILINGQNIREVDLQSLRRAIAVVPQDSVLFHDTIRHNIHYGDLSKSQEELENAARMADLHESIQQW  700
AGAP006364      IPAGKKIAIVGGSGSGKSSMVRLLFRFFEPSTGQILINGQNIREVDLQSLRKAIAVVPQDSVLFHDTIRHNIHYGDLSKSQTELENAARMADLHDSILQW
                ************************:*****:**:*****************:***************************** ************:** **

AnstABCB4       PKQYDTQVGERGLKLSGGEKQRVAIARAILKNSPILIFDEATSSLDSITEHNILQALARATDNRTSICIAHRLSTVMDADEILVLENGRVAQRGTHDQLL  800
AGAP006364      PKQYETQVGERGLKLSGGEKQRVAIARAILKNSPILIFDEATSSLDSITEQNILQALARATDNRTSICIAHRLSTVMDADEILVLENGRVGQRGTHDQLL
                ****:*********************************************:***************************************.*********

AnstABCB4       RSGGLYTKLWDTQNRLYNIGGKKLNAEEKK--  831
AGAP006364      RSGGLYTKLWDTQNRLYNIGSKKPTKPEGEEK
                ********************.** .  * :
```

AnstABCBmember6

```
AnstABCBmember6  MIHYCPTNTSMNVVWFNHGVSQCFMDTVAMGTIGGFMLIFGTLQLLMYLRHATEIDTQRIRKSRLYNFQLFLLLLMPLLTVARFVLEGFI   90
AGAP002278       MIRYCPTNTSMDVVWFNHGVSQCFMDTVAMGTIGGFMLIFGTLQLIMYLRHATEIDTQRIRKSRLYNFQLFLLVLMPLLTTARFVLEAFV
                 **:********:*********************************:***************************:******.******.*:

AnstABCBmember6  FDGAQVYGFMVLSIAVALFAYPFAIVLLVKERYYQLPSVPTRGHGLVLLLFWTLVFIVQNIAFINLNYHDAWFRLETLRDKVEFGMFVAR  180
AGAP002278       FEGAQVYGFMVLSIAVALFAYPYSIVLLVKERYYQLPSVPTRGHGLVLLVFWTLLFIVQNIAFVNLNYRDAWFRLETVRDRVEFGMFVAR
                 *:********************::*************************:****:********:****:********:**:*********

AnstABCBmember6  YTITMLLFVIGLKAPGITSTQFTEEYQNLHQAQ-ENQSTFSNAWTKMRTLLPFLWPKKDAFLQFRVVFCFLLLIAGRLINVYVQIYNKKI  270
AGAP002278       YTVTMLLFVIGLKAPGITSTQFTEEYQHLHQAQ-ENRSTFANAWTKMRTLLPFLWPKKDALLQFRVVFCFLLLIAGRLINVYVQIYNKKI
                 **:************************:***** **:***:*******************:*****************************

AnstABCBmember6  VDSLTEKPATFRWDWILLYVGFKFLQGGGTGSMGLLNNLRSFLWIRIQQYTTREIELELFRHLHSLSLRWHLNRKTGEVLRVMDRGTDSI  360
AGAP002278       VDSLTEKPTVFRWDWILLYVGFKFLQGGGTGSMGLLNNLRSFLWIRIQQYTTREIELELFRHLHSLSLRWHLNRKTGEVLRVMDRGTDSI
                 ********:.********************************************************************************

AnstABCBmember6  NNLLNYILFSITPTIVDILIAVVFFITAFNWWFGFIVFLTMTLYIVATIMVTEWRTKFQRRMNLADNQQKARSVDSLLNFETVKYYGAEQ  450
AGAP002278       NNLLNYILFSITPTIVDILIAVVFFITAFNWWFGFIVFLTMTLYIVATIMVTEWRTKFQRRMNLADNQQKARSVDSLLNFETVKYYGAEQ
                 ******************************************************************************************

AnstABCBmember6  YEVSCYRDAILKFQDEEWRSIITLNILNTMQNVIVCGGLLAGSMLCAYLVVYEEGLTVGDYVLFASYIIQLYVPLNWFGTFYRAIQKNFV  540
AGAP002278       YEVSCYRDAILKFQDEEWRSIITLNILNTMQNIIVCGGLLAGSLLCAYLVVYEEGLTVGDYVLFASYIIQLYVPLNWFGTFYRAIQKNFV
                 ********************************:**********:**********************************************

AnstABCBmember6  DMENMFDLMREEQEVLDAPSAPGLAVARGRIDFNDVTFGYNAERLVLRNVSFTVPGGKTVAIVGPSGAGKSTIMRLLFRFYDVNSGSISV  630
AGAP002278       DMENMFELMREDQEVVDAPSAPGLAVARGRIDFNDVTFGYNAERLVLRNVSFSVPGGKTVAIVGPSGAGKSTIMRLLFRFYDVNNGSILV
                 ******:****:***:************************************:*******************************.*** *

AnstABCBmember6  DGQNIKIVRQASLRQAIGVVPQDTVLFNNTIKYNIQYGRVGAPEADVIMAARSADIHERILTFPEQYETQVGERGLRLSGGEKQRVAIAR  720
AGAP002278       DGQNIKTVRQASLRQAIGVVPQDTVLFNNTIKYNIQYGRVGAPEADVIMAARSADIHERILTFPEQYETQVGERGLRLSGGEKQRVAIAR
                 ****** ***********************************************************************************

AnstABCBmember6  TILKSPAIVLLDEATSALDTQTERNIQTALAKVCANRTTIIIAHRLSTIIHADEILVLKEGSIVERGRHEMLLDQNGVYAEMWNQQLKNL  810
AGAP002278       TILKSPAIVLLDEATSALDTQTERNIQSALAKVCANRTTIIIAHRLSTIIHADEILVLKDGSIVERGRHDNLLEQSGVYAEMWNQQLKNL
                 ***************************:*******************************:*********: **:*.**************
```

(continue)

(continue)

```
AnstABCBmember6  ELGASATEEAALVDGNGQVANGTPNKLPAIAPPNHQHHPHH  851
AGAP002278       ELGGSGAAEEGVDAANGQVANGTPNKVAAIAAPPAHHHHH-
                 ***.*.: * .:  .***********:.***.*  :** *
```

AnstABCG4

```
AnstABCG4      DKDQENLSVASSGFGYSKTYEVVVLLHFQQANKTSKEGSAGPRDGQKMVGLKNKQLINFLSSCDFCFRFRLASAFFAYRPLVPYLHFEGFFLWLFSIELI  100
AGAP001333     ----------------------------------------------------------------------------------------------------

AnstABCG4      LCFVQFYLLPALPKRTAGVFRYGKGAHLLNDLFYGILGNKRLVVFETILRVFVELDHFGVREARASASVMSTWETIANESWAVVDNLDGMPPVAGRSSTD  200
AGAP001333     ---------------------------------------------------------MSAREAVVSTAVCCTVDNLDGMPGVTVAQHE---VAAAAADDD
                                                                        :..*** .*::* .* :.: . . ..* : :    .*. :. *

AnstABCG4      VTLMWQNLTITPVRSTPSSGGGGQLPVLNDISGTLQPGTLVALMGPSGAGKTTLMSALAYRMSDKMTITGDIRVNGCPIGPFMYNISGYIYQDELLPDSI  300
AGAP001333     VTLIWQNLTITPIRSKP--GAGEQHPVLNDISGTLQPGTLVALMGPSGAGKTTLMSALAYRMSDKMTIAGDIRVNGCPIGPYMYNISGYIYQDELLPDSI
               ***:********:**.*  *.* * *******************************************:************:******************

AnstABCG4      TVQEHLQLMAHLKLGKRVSADRKRTMIADILSKTGLERCANTKIANASGIGKTLSGGEKKRLAFAVELLSKPKFLFCDEPTTGLDSYSARQLVEMMKSLT  400
AGAP001333     TVQEHLQLMANLKLGKSVTAERKRAMIAHILSRTGLERCANTKIADATGIGKTLSGGEKKRLAFAVELLSKPKFLFCDEPTTGLDSYSARQLVEMMKSLT
               **********:***** *:*:***:***.***:************:*:****************************************************

AnstABCG4      RAGTTVLCSIHQPAEKLLYEFDSLILLTGGQTGFIGSPHEAVQFFRLQGLECEHGYNTADFLIKVLSSTTTAKTTKGNQFTTGAICPKTICNNYIASEAA  500
AGAP001333     RTGTTVLCSIHQPAEKLLYEFDSLILLTGGRTGFIGAPSEAVQFLRLQGLECEAGYNTADFLLKVLSSTTTT-TRKGNQFTTGAIGPKTICNNYSASEAA
               *:****************************:*****:* *****:******** ********:********: * ********** ******** *****

AnstABCG4      RRQEALISAELYSTAVD-GRDEAFRKQLTKARDRCWFYTLYWLMYRHVLQAHRNPNLQYFKIAQRIAIAVLVGLCFSDAIELSQRGVQAMQGVIFLIVSE  600
AGAP001333     RRQEALISVELYRTTVDSGGDEAFRRRLTESRDRCWFYTLYWLMYRHVLQSHRNPNLQYFKIVQRIAIAVLVGLCFSDAIELSQRGVQAMQGVIFLIVSE
               ********.*** *:** * *****::**::*******************:***********.*************************************

AnstABCG4      NTFLPMYAALSMFPDSFPLFQREKKANLYSTAQFYISSILSMSPFVLLETCAFILIVYFLANLRPTLLGLIVTVAVSVLVMNVSIACGCFFSTLFPTVAS  700
AGAP001333     NTFLPMYAALSLFPERFPLFQREKKANLYSTAQFYISTIMSMTPFVLLETCAFILIVYFLANLRPTLLGLLVTVVVSVLVMNVSIACGCFFSVLFPTVAS
               ***********:**: *********************:*:**:***************************:***.*****************.*******

AnstABCG4      AMSYLVPFDYILMITSGIF-  720
AGAP001333     AMSYLVPFDYILMITSGIFI
               *******************
```
