# Supplementary material for: ABC transporters are involved in defense against permethrin insecticide in the malaria vector Anopheles stephensi
Source: Parasit Vectors. 2014 Jul 29;7:349. doi: 10.1186/1756-3305-7-349 (PMC4124152; doi:10.1186/1756-3305-7-349)
Supplement: Supplementary file 3 — Additional file 3: Table S2: Dayhoff PAM matrix. Estimates of distance among the ABC transporters identified in Anopheles stephensi and the homologous ABC transporters of other mosquitoes species are shown: An. gambiae (AGAP005639; AGAP006273; AGAP006364; AGAP002278; AP001333), An. darlingi (ETN61204; ETN66919; ETN62617; ETN64062; ETN58714), Aedes aegypti (AAEL010379; AAEL002468; AAEL006717; AAEL008134; AAEL003703), and Culex quinquefasciatus (EDS44274; EDS35382; EDS29700; EDS27088; EDS37204). (DOC 15 KB) [file 13071_2014_1536_MOESM3_ESM.doc]

**Table S2.** **Dayhoff PAM matrix.** Estimates of distance among the ABC transporters identified in *Anopheles stephensi* and the homologous ABC transporters of other mosquitoes species are shown: *An. gambiae* (AGAP005639; AGAP006273; AGAP006364; AGAP002278; AGAP001333)*, An. darlingi* (ETN61204; ETN66919; ETN62617; ETN64062; ETN58714)*, Aedes aegypti* (AAEL010379; AAEL002468; AAEL006717; AAEL008134; AAEL003703)*,* and *Culex quinquefasciatus* ([EDS44274](http://blast.ncbi.nlm.nih.gov/Blast.cgi#alnHdr_170063163); [EDS35382;](http://blast.ncbi.nlm.nih.gov/Blast.cgi#alnHdr_170063163) EDS29700; [EDS27088](http://blast.ncbi.nlm.nih.gov/Blast.cgi#alnHdr_170042892); EDS37204)*.*

|  | *Anst*ABCB2 | *Anst*ABCB3 | *Anst*ABCB4 | *Anst*ABCBmemb6 | *Anst*ABCG4 |
| --- | --- | --- | --- | --- | --- |
| *Anopheles gambiae* | 0.0578 | 0.0610 | 10.5138 | 0.0641 | 12.2969 |
| *Anopheles darlingi* | 0.1152 | 0.2125 | 14.1945 | 2.5713 | 15.0904 |
| *Aedes aegypti* | 0.1973 | 0.3705 | 12.9521 | 8.9150 | 39.1189 |
| *Culex quinquefasciatus* | 0.2573 | 0.3418 | 39.3436 | 10.7088 | 45.3775 |
